# Mental fatigue and sleep restriction effects on perceptual-cognitive performance in trained beach volleyball athletes

**DOI:** 10.3389/fpsyg.2025.1537482

**Published:** 2025-05-09

**Authors:** Bruno Teixeira Barbosa, Dalton de Lima-Junior, Alexandre Moreira, Fábio Yuzo Nakamura, Gilmário Ricarte Batista, Heloiana Faro, Leonardo de Sousa Fortes

**Affiliations:** ^1^Grupo de Pesquisa em Neurociência e Desempenho Esportivo, Department of Physical Education, Federal University of Pernambuco, Recife, Brazil; ^2^Graduate Associate Program in Physical Education, Federal University of Paraiba, João Pessoa, Brazil; ^3^Graduate Program in Physical Education, Federal University of Pernambuco, Recife, Brazil; ^4^Department for Life Quality Studies, University of Bologna, Bologna, Italy; ^5^Department of Sport, School of Physical Education and Sport, University of São Paulo, São Paulo, Brazil; ^6^Department of Physical Education and Sport Sciences, Maia University Institute, Maia, Portugal; ^7^Department of Physical Education, Federal University of Paraiba, João Pessoa, Brazil

**Keywords:** cognitive fatigue, partial sleep deprivation, athletic performance, brain, team sports

## Abstract

**Introduction:**

Perceptual-cognitive skills are essential in sports such as beach volleyball. Mental fatigue (MF) and sleep restriction (SR) are known to impair athletes’ perceptual-cognitive performance. This study aimed to analyze the effects of MF and SR on perceptual-cognitive performance in beach volleyball athletes.

**Methods:**

Fourteen volunteers participated (12 men; 17.9 ± 1.2 years; 182.0 ± 7.7 cm; 72.3 ± 8.4 kg) and were randomly submitted to four experimental conditions: (a) Control (CT), (b) MF, (c) SR, and (d) SR + MF. The MF cognitive load and SR duration were individualized in MF and SR experimental conditions. Perceptual-cognitive performance was assessed through two visuomotor tests utilizing LED light technology to simulate defensive and blocking actions within a real beach volleyball context. Visuomotor tests assessed reaction time (RT) measures. The Generalized Linear Models were applied to verify the main effects of the conditions for the perceptual-cognitive responses.

**Results:**

The results showed a significant condition effect for both the defense [*X*^2^_(3,39)_ = 212.2; *p* < 0.001] and block tests [*X*^2^_(3,39)_ = 104.0; *p* < 0.001]. In the defense test, participants in the CT condition had significantly faster RTs (1727.0 ± 113.0 ms; *p* < 0.001) compared to all other conditions (MF: 1806.0 ± 126.0 ms; SR: 1874.0 ± 145.0 ms; and SR + MF: 1906.0 ± 133.0 ms). Additionally, the SR and SR + MF conditions showed slower RTs than MF (*p* = 0.004 and *p* < 0.001, respectively), with no significant difference between SR and SR + MF (*p* = 0.41). In the block test, the CT condition also resulted in faster RTs (631.0 ± 82.2 ms; *p* < 0.001) compared to MF (711.0 ± 77.5 ms), SR (691.0 ± 82.3 ms), and SR + MF (722.0 ± 100.0 ms). SR had better RTs than MF (*p* = 0.04) and SR + MF (*p* = 0.01), with no differences between MF and SR + MF (*p* = 0.61).

**Conclusion:**

Therefore, we conclude that both the isolated and combined effects of MF and SR negatively impact RT in the defense and block visuomotor tests. This suggests that MF and SR conditions may impair athletes’ ability to respond quickly to visual stimuli in different scenarios.

## 1 Introduction

Perceptual-cognitive skills are crucial in numerous sports and involve the capacity to perceive, interpret, and react to stimuli as quickly as possible (Hodges et al., 2021). Within this context, perceptual-cognitive skills encompass a spectrum of abilities ranging from sensory detection to the integration of environmental cues with prior knowledge, facilitating appropriate responses (Williams and Ericsson, 2005). In beach volleyball, this is exemplified by the player’s ability to locate, track, and respond to both opponent actions and the trajectory of the ball, requiring perceptual acuity from the athlete (Hodges et al., 2021; Jafarzadehpur et al., 2007; Vansteenkiste et al., 2014). Given that, visual attention is considered a subset of perceptual-cognitive skills and reflects the speed of processing information, commonly measured as reaction time (Hodges et al., 2021). In beach volleyball, where visual perception predominates, players must observe the ball and opponents’ body language attentively and anticipate their actions (Williams and Ericsson, 2005; Jafarzadehpur et al., 2007; Vansteenkiste et al., 2014). Notably, these skills are not solely confined to perception and cognition but are deeply intertwined with motor responses in response to opponents’ actions (Hodges et al., 2021). In particular, in actions like defense and block, players must quickly perceive and interpret the opponent’s movements and the ball’s trajectory to make an effective response. These actions require not only speed but also the ability to anticipate and react accordingly. Thus, in dynamic and unpredictable sports such as beach volleyball, perceptual-cognitive skills represent the combination of sensory perception, cognitive processing, and motor execution that might influence performance outcomes. In this sense, perceptual-cognitive skills are typically sensitive to mental fatigue (MF) and sleep restriction (SR) and they are often impaired in athletes experiencing MF (Cao et al., 2022; Habay et al., 2021a) and SR (Charest and Grandner, 2020; Fullagar et al., 2023; Walsh et al., 2021).

Given that, MF is a state of psychobiological tiredness, lack of energy, lethargy, and a higher-than-normal perception of effort caused by periods of demanding cognitive activities such as computerized tests (Gantois et al., 2020), smartphone use (Fortes et al., 2019) or playing videogames (Fortes et al., 2022a), while SR is associated with sleep loss, it differs from sleep deprivation (SD) as it refers to partial SD, while SD refers to total SD (≥24 h without sleep) (Fullagar et al., 2023).

Previous studies have investigated the isolated effects of MF and SR on athletes’ perceptual-cognitive performance (Romdhani et al., 2019; Jarraya et al., 2014). Both conditions appear to impair performance through psychobiological mechanisms. In the case of MF, an increased perception of effort is attributed to adenosine accumulation in the prefrontal and anterior cingulate cortices (Martin et al., 2018). On the other hand, SR impacts performance by disruptions in prefrontal cortex functioning, reducing cerebral metabolism in the thalamus and cerebellum, influencing homeostatic sleep regulation, and affecting the circadian pacemaker (Fullagar et al., 2023). In real-world training and competition scenarios, athletes are often exposed to both MF and SR simultaneously, which may exacerbate performance deficits, including an increased likelihood of errors, diminished capacity to track and respond to opponent actions and ball trajectory, and compromised decision-making abilities. These impairments may also affect the ability to process information or feedback from coaches and teammates, further impacting performance under high-pressure conditions (Habay et al., 2021a; Fullagar et al., 2023).

However, the combined effects of MF and SR on athletes’ performance have not been thoroughly examined. Among the few studies that have combined the effects of MF and SR on athletes’ performance, Filipas et al. (2021a) observed an impairment (≈7%) in free-throw performance in basketball players by MF and SR combined, similar to MF and SR alone. In this study, the MF condition was induced by tactical basketball videos, and young basketball players were allowed to sleep for only 5 h, with SR being conducted by going to bed later than usual. However, the SR duration was not individualized in the study, and only two experimental conditions were performed. That individualization is essential once 3 h of SR, for example, might represent different relative SR times/loads depending on the habitual total sleep duration of each subject. Supporting this notion, studies by Fullagar et al. (2023) and Habay et al. (2021a) further emphasize that both SR and MF, when examined separately, have been shown to impair sport-specific and cognitive performance in athletes from various sports, although the findings are still conflicting. More research is needed to understand the combined and solo effects of SR and MF on athletes’ performance. Nevertheless, the combined negative effects of SR and MF on athletes’ performance are still unclear in the literature, and further investigation may clarify athletes’ behaviors and assist coaches’ decision-making during training and matches.

Despite recent studies on MF, SR, and their effects on athletes’ performance (Habay et al., 2021a; Fullagar et al., 2023), none have individualized the cognitive-demand task load to induce MF or assessed habitual sleep duration to personalize the duration of SR when evaluating their isolated and combined effects on perceptual-cognitive performance in team-sport athletes. Additionally, performance under MF and/or SR conditions in athletes is commonly assessed through laboratory experiments rather than field ones. Therefore, this study aims to evaluate the isolated and combined effects of MF and SR on perceptual-cognitive performance in beach volleyball athletes using visuomotor tests that simulate an ecological beach volleyball context.

## 2 Methods

### 2.1 Participants

**Figure 1:**
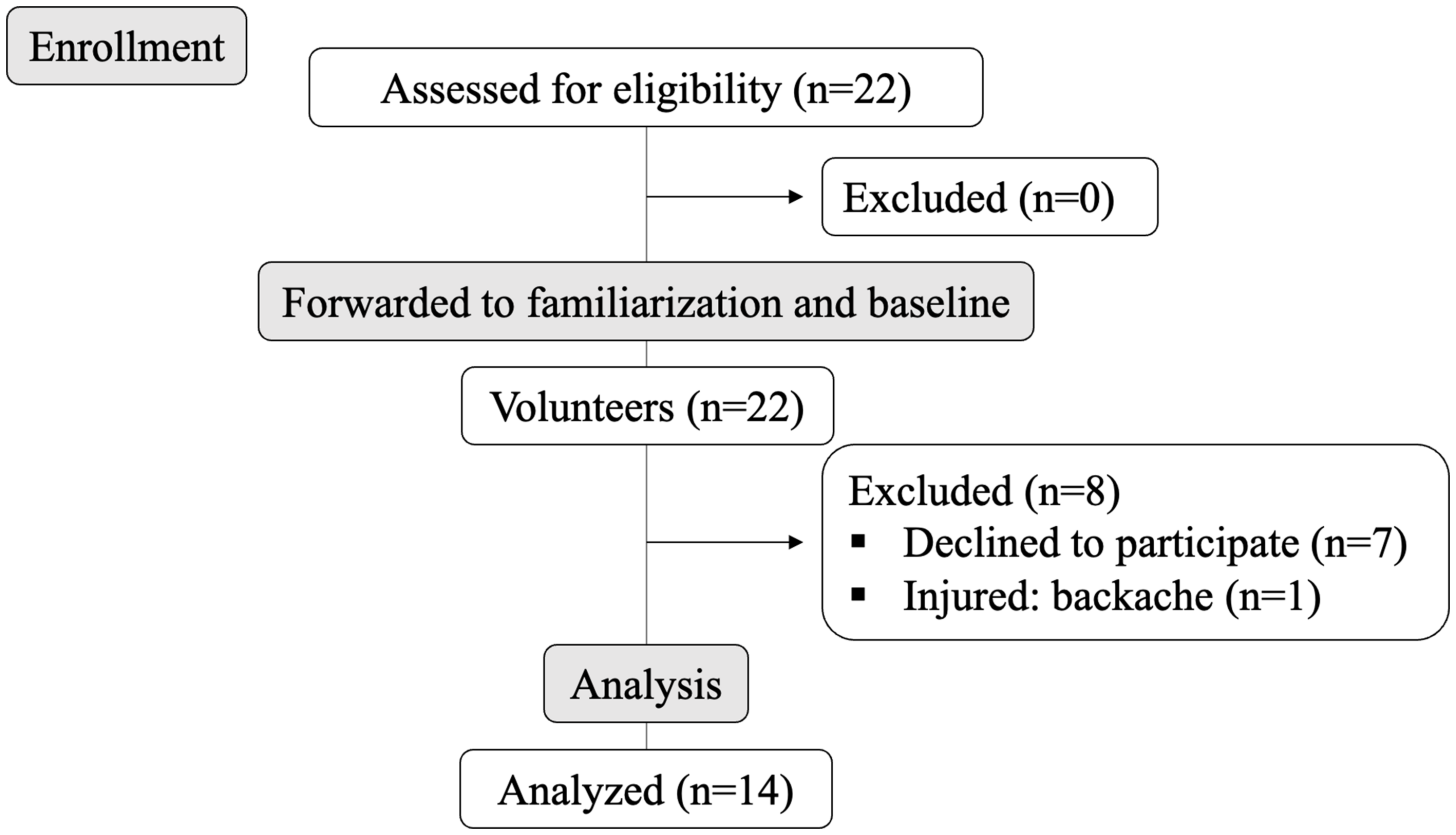
Flow diagram of volunteer participation throughout the study.

Fourteen beach volleyball players from different competitive levels (State, *n* = 1; Regional, *n* = 5; National, *n* = 8) were enrolled in the study (age: 17.92 ± 1.24 years; height: 182.0 ± 7.7 cm; weight: 72.3 ± 8.4 kg; BMI: 21.93 ± 1.45 kg/m^2^; Defense, *n* = 7; Block, *n* = 7; years of experience: 3.0 ± 1.4; training sessions per week: 5.0 ± 1.0; hours of training per day: 2.3 ± 0.7; Under 17, *n* = 3; Under 19, *n* = 3; Under 21, *n* = 8). Participants were recruited through digital media, social networks, and beach volleyball training centers in João Pessoa/PB/Brazil. Participants should be involved in beach volleyball for at least 1 year, maintain a systematic training routine, and be able to perform tests and protocols without any restrictions. Besides, participants were excluded if they were color blind or had injuries that could compromise their ability to move quickly in different directions during the tests. Written informed consent was obtained from all participants or their parents/legal guardians (for minors), and the study was approved by the university research ethics committee and followed the Declaration of Helsinki. The flow diagram of volunteer participation, from enrolment to data analysis, is shown in Figure 1.

### 2.2 Experimental design

**Figure 2:**
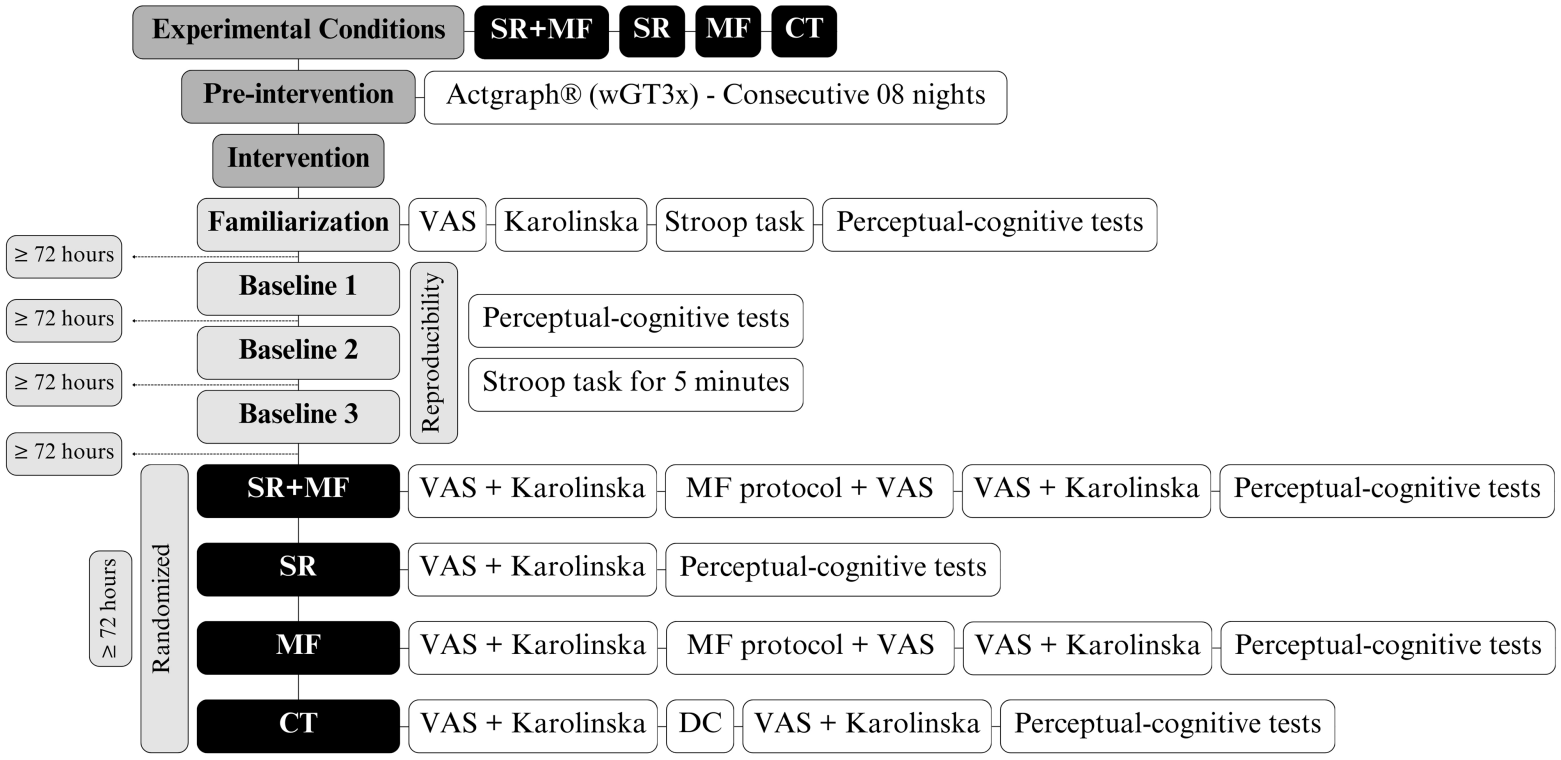
Study design. SR, sleep restriction; MF, mental fatigue; CT, control; VAS, visual analog scale; DC, documentary.

This is a repeated-measures experimental design study with simple randomization of the experimental conditions (see Figure 2). A 72-h washout was implemented between each condition to prevent residual effects. The study assessed perceptual-cognitive performance through reaction time measures. Data collection occurred in the morning in three different environments: (1) The Stroop task and MF assessment were conducted in an air-conditioned lab (≅21°C), (2) and visuomotor tests were conducted on a sand court. The study consisted of four experimental conditions [1. SR + MF, 2. SR, 3. MF, and 4. Control (CT)], with volunteers watching a neutral documentary for 45 min in the CT condition. Familiarization session was conducted once, while baseline sessions were conducted three times to avoid learning effects and to assess test–retest reliability. In the experimental conditions with SR, volunteers were instructed to arrive at the lab within 1 h of waking in the morning, based on their individual sleep behavior.

### 2.3 Stroop task and MF assessment

The computerized version of the Stroop task in Psychopy software (version 2020.2.4) was used to induce MF (Stroop, 1935). The task was displayed on a 20-inch screen (HP Compaq, LA2006x, 16,000×900) using incongruent stimuli plus a switch function with the colors/words “yellow,” “blue,” “green,” and “red.” Volunteers responded with the keys “A” for “yellow,” “D” for “blue,” and “J” for “green,” and adhesive tapes of respective colors were placed on the keyboard. Volunteers were encouraged to use their hands (left/right) to perform the task in the way they felt most comfortable (see Supplementary Figure 1).

Volunteers were encouraged to indicate the word colors and ignore its meaning. The “switch” function presented the words “yellow,” “blue,” or “green” in red, requiring volunteers to associate the motor response with the word’s meaning rather than its color. The stimuli appeared in a random 100% incongruent sequence, with each word displayed for 1,000 ms and followed by 1,000 ms of feedback before the next word. The task lasted at least 30 min and continued until the volunteer pointed twice, consecutively or not, at least 70 mm on the Visual Analog Scale (VAS). The VAS was responded to every 5 min to monitor MF state.

Accuracy and response time were analyzed in five-minute blocks to serve as behavioral indicators of MF. The accuracy percentage was analyzed, and only correct responses were considered for the response time analysis. The intraclass correlation coefficient (ICC) for the Stroop task RT was 0.89, obtained after two baseline meetings.

### 2.4 Visuomotor tests

The visuomotor tests, which simulated blocking and defending situations in beach volleyball, used stationary LED lights (ReactionX®, United Kingdom). The tests were performed in an open environment without rain, sunglasses or a cap. Supplementary material shows the visual set for visuomotor tests.

#### 2.4.1 Visuomotor block test

The test assessed mean RT using the “Find difference” mode on the ReactionX® app (see Supplementary Figure 2). Three LED lights were placed 1.60 m high with a 50 cm horizontal distance between them, and the volunteer stood 60 cm from the central light. The volunteer was instructed to position their hands in anticipation of blocking, keeping their elbows close to their body and blocked the light with their hands in anticipation of the test. The test used 86 stimuli, based on the average number of jumps in blocking actions reported in a previous study (Turpin et al., 2008). The LED lights, consisting of a set of three lamps with one target and one false color, turned on randomly within a delay of 0.5 to 3.0 s. If the volunteer took more than 3 s to respond, the test continued, and the stimulus counted as a loss. No stimuli other than visual cues were used, and the ICC for the test was 0.88, obtained after three baseline meetings (see Supplementary Figure 3). The visuomotor block test lasted an average of 3 min and 40 s in this study.

#### 2.4.2 Visuomotor defense test

This test evaluated mean RT in three simulated defense situations in beach volleyball: (Hodges et al., 2021) cut shot, (Williams and Ericsson, 2005) poke shot, and (Jafarzadehpur et al., 2007) long shot. Four LED lights were positioned on the sand, suspended by rods that were 60 cm high, with 20 cm buried in the sand (see Supplementary Figure 4). The LED lights were positioned 40 cm from the ground to prevent sensor activation by displaced sand from the athlete’s movements during the test. One LED light served as the base, with the other three positioned three meters away from the base for displacement and recording of the volunteer’s RT. The athletes were required to turn off the light using only their hands. The athlete stood 60 cm from the base pole and positioned their left or right leg in front of the bar. The “Homebase” mode was used on the ReactionX®, with four lamps, and the athlete was asked to move to LED lights 1, 2, or 3 as quickly as possible. LED lights 1, 2, and 3 were randomly activated with a delay of 0.5 to 3.0 s for turning on the lights, with the base light serving as a trigger for the other lights (see Supplementary Figure 5). No stimuli other than visual cues were used, and the time interval for turning on the LED lights was random. Only the green color was used, as this was the most prominent color in an open environment. Each volunteer completed the test by responding to a total of 20 stimuli. The ICC was 0.88, obtained after three baseline meetings. The visuomotor defense test lasted an average of 3 min and 5 s in this study.

### 2.5 Sleep behavior, sleep restriction, and sleepiness

Volunteers’ sleep behavior was monitored over eight nights using Actgraph® wGT3x, a device positioned on the non-dominant wrist. The first night of monitoring was excluded from analysis to account for the volunteers’ familiarization with the instrument. Data were collected at a frequency of 30 Hz and analyzed in 60-s epochs, with sleep and wakefulness evaluated using the Sadeh algorithm (Sadeh, 2011) and Actilife software version 6.13.3. This analysis provided information such as sleep latency, sleep start and end time, sleep duration time, time in bed, and sleep efficiency. A digital sleep diary (online form) was also created for volunteers to report when they went to bed and woke up in the morning.

A 50% SR was implemented among the volunteers based on the sleep duration time of each volunteer. To monitor SR, volunteers completed an online form at 15-min intervals from when the SR period began until the SR period was completed. Volunteers were instructed to go to bed later than usual rather than waking up earlier. This approach was chosen as social demands, like smartphone use, are common non-sport factors linked to sleep disturbances in athletes (Walsh et al., 2021). Given the increasing use of technology, this method may better reflect real-world conditions than assessing SR at the end of the night (Mei et al., 2024). For instance, the volunteer who was instructed to restrict themselves of 4 h of sleep (from 11:00 p.m. to 03:00 a.m.) should fill out the online form from 11:15 p.m. until 03:00 a.m. at 15-min intervals (16 times). If the form was not adequately completed, the experimental condition would have to be rescheduled.

The Karolinska Sleepiness Scale (KSS) was used to assess subjective sleepiness (Åkerstedt and Gillberg, 1990). A modified version of the KSS with a scale ranging from 0 to 10 was used (Shahid et al., 2012). KSS was applied before getting sleep and after waking up, in addition to pre-MF induction, every 5 min during MF induction, and post-MF induction throughout each experimental condition.

### 2.6 Power calculation

We calculated the power of the study using the app GLIMMPSE.^1^ The calculus was made as follows: (a) target power of 0.8; (b) statistical test was Hotelling Lawley Trace; (c) type one error rate of 0.05; (d) primary outcome being perceptual-cognitive responses; (e) repeated measures for condition (categorical) with four measurements (CON × MF × SR × SR + MF); (f) no clustering was add in the analysis; (g) no fixed predictors; (h) hypothesis choice as the main effect of condition; (i) hypothesis contrast of all mean differences zero; (j) theta of zero; (k) group size ratio of 1-1; (l) marginal means based on the results of our study; (m) scale factor for the marginal of 1; (n) variability across outcomes of 0.3; (o) repeated measure standard deviation ratios of 1 for the conditions; (p) repeated measure correlation with an unstructured matrix; and (q) scale factor variance of 1. We estimated a power of 0.90 for the sample size of 14 participants.

### 2.7 Statistical analysis

The study’s power was calculated using the GLIMMPSE app (see Footnote 1) with a target power of 0.8 and a type I error rate of 0.05. The primary outcome was perceptual-cognitive responses, analyzed using Hotelling’s Lawley Trace with four conditions (CON, MF, SR, SR + MF). No clustering or fixed predictors were included, and an unstructured correlation matrix was used. Based on a sample size of 14 participants, the estimated power was 0.90.

The Generalized Mixed Models (GLzMM) analyzed the main effects and interaction between conditions (i.e., CON × MF × SR × SR + MF) and time (i.e., pre × post) for the subjective and behavioral outcomes. The Generalized Linear Models (GLzLM) were applied to verify the main effects of the conditions for the perceptual-cognitive responses. The GLzMM set up as follows: (a) subjects, group, time, and interaction were tested in the model as random effects; (b) time as the within-subject variable; (c) Gamma or Gaussian distributions with identity link function for model type; (d) condition and time as factors; (e) Akaike Information Criterion for the better-fit model; (f) Wald chi-square statistics as the model effects; (g) Holm post-hoc for pairwise comparisons; and (h) graphic analysis of the residuals (Zeger and Liang, 1986). The data are presented in mean and 95% confidence interval of the mean. In case of significant results, the 95% confidence interval of the mean differences (CI95%diff) is presented. The analyses were made using JAMOVI v2.5.3.0.

## 3 Results

The 50% SR in this study represented an overall restriction of 201 ± 15 min (≈3 h and 20 min). In addition, Table 1 describes the sleep profile of the volunteers at baseline.

**Table 1:** Sleep profile of the volunteers.

Variables	Subjects (*n* = 14)
Sleep latency, min	8.99 ± 6.35
Sleep efficiency, %	80.39 ± 3.91
Total time in bed, min	515.95 ± 49.19
Sleep total time, min	416.70 ± 49.22

### 3.1 Subjective responses of MF and sleepiness to the Stroop task and the documentary

#### 3.1.1 Visual analog scale

**Figure 3:**
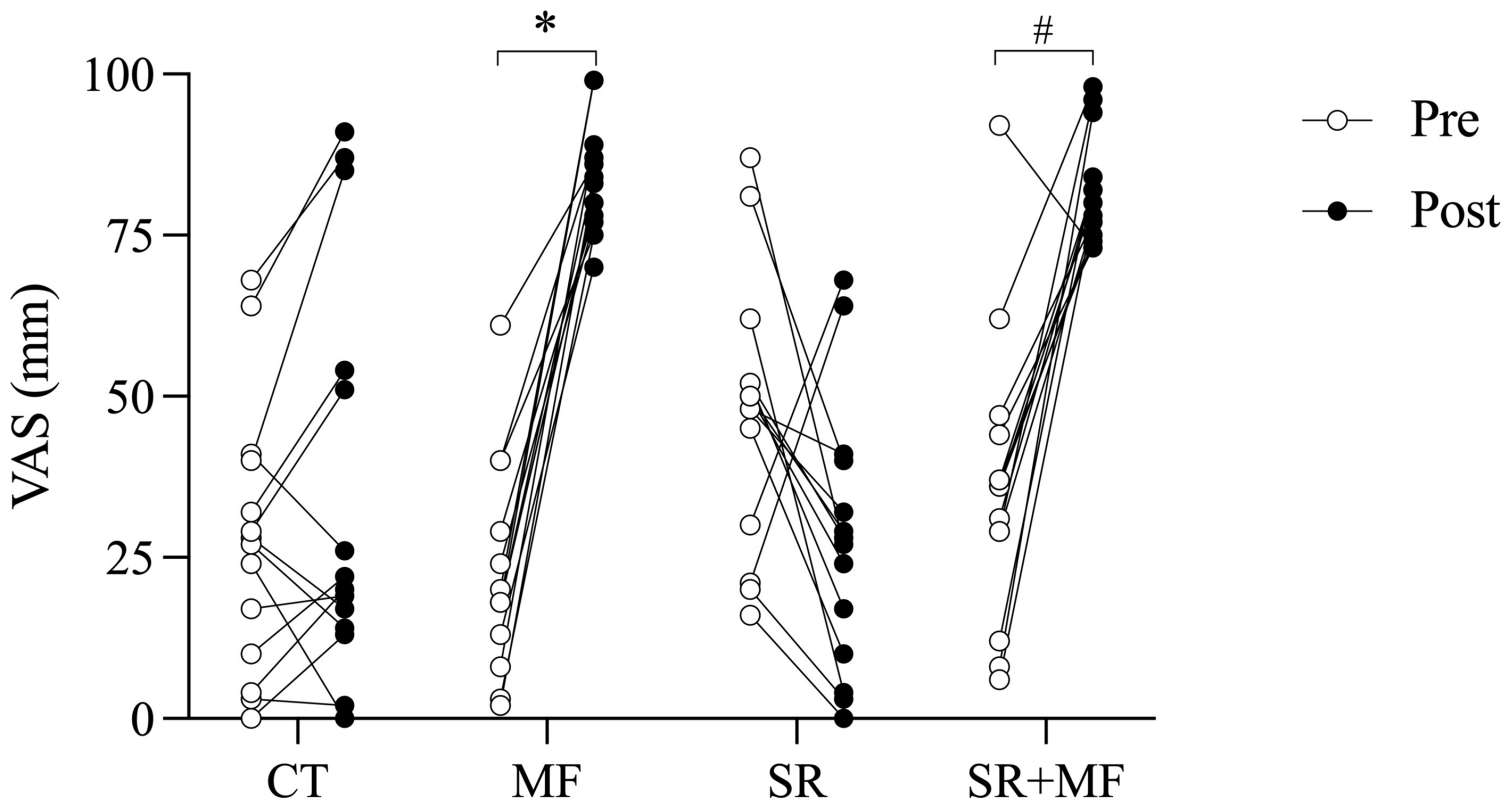
Subjective responses of MF to the experimental conditions. VAS, Visual Analog Scale; CT, control; MF, mental fatigue; SR, sleep restriction; SR + MF, sleep restriction + mental fatigue. Comparison between pre- and post-MF induction protocols (Stroop Task and Documentary) within the MF (^*^p < 0.001) and SR + MF (^#^*p* < 0.01) experimental conditions.

A condition × time interaction was observed [*F*_(3,91)_ = 30.0; *p* < 0.001; ηp^2^ = 0.78; large effect] for MF in trained athletes submitted to the Stroop task. No statistically significant differences were observed in the pre-MF induction VAS between the experimental conditions (*p* > 0.05). The documentary in the CT condition (pre-CT vs. post-CT: 24.3 ± 18.9 mm vs. 33.3 ± 30.3 mm; CI95%_diff_ = −8.1 (−28.9 to 12.6); *p* = 1.00) did not result in changes in the VAS; however, statistically significant increases in VAS were observed after the Stroop task for the MF (pre-MF vs. post-MF: 22.1 ± 17.2 mm vs. 83.7 ± 9.1 mm; CI95%_diff_ = −60.7 (−81.5 to −40.0); *p* < 0.001; ↑ 279%; Cohen’s d: 3.26; strong effect) and SR + MF (pre-SR + MF vs. post-SR + MF: 36.2 ± 22.2 mm vs. 81.7 ± 8.5 mm; CI95%_diff_ = −45.5 (−66.2 to −24.8); *p* < 0.01; ↑ 126%; Cohen’s d: 1.79; strong effect) conditions (Figure 3).

#### 3.1.2 Karolinska

A main effect of condition [*X*^2^_(3,91)_ = 30.5; *p* < 0.001] and time [*X*^2^_(3,91)_ = 51.3; *p* < 0.001] were found. However, the results lacked interaction [*X*^2^_(3,91)_ = 1.9; *p* = 0.60].

### 3.2 Subjective responses of MF and sleepiness to experimental conditions

#### 3.2.1 Visual analog scale

We found a condition effect [*F*_(3,91)_ = 6.8; *p* < 0.001], but time [*F*_(1,91)_ = 2.4; *p* = 0.12] and interaction [*F*_(3,91)_ = 0.4; *p* = 0.74] were not significant.

#### 3.2.2 Karolinska

We found a condition effect [*F*_(3,91)_ = 5.5; *p* = 0.002], but no time [*F*_(1,91)_ = 1.3; *p* = 0.25] and interaction [*F*_(3,91)_ = 0.1; *p* = 0.96] effects were found.

### 3.3 Behavioral responses to the Stroop task

#### 3.3.1 Response time

No condition [*F*_(1,26)_ = 0.5; *p* = 0.50], time [*F*_(1,13)_ = 1.3; *p* = 0.27] and interaction [*F*_(1,26)_ = 0.6; *p* = 0.45] effects were found.

#### 3.3.2 Accuracy

We found a condition [*X*^2^_(1,39)_ = 5.8; *p* = 0.02], time [*X*^2^_(1,39)_ = 9.9; *p* = 0.002] and interaction [*X*^2^_(1,39)_ = 4.9; *p =* 0.03] effects. While MF condition showed pre- and post-similar results [pre-MF vs. post-MF: 94.2 ± 3.4% vs. 93.7 ± 3.8%; CI95%_diff_ = 0.5 (−1.7 to 2.5); *p* = 0.99], SR + MF condition presented different accuracy values comparing pre-and post-Stroop task [pre-SR + MF vs. post-SR + MF: 96.4 ± 2.1% vs. 93.8 ± 2.4%; CI95%_diff_ = 2.6 (0.5 to 4.7); *p* < 0.001].

### 3.4 Perceptual-cognitive responses to the visuomotor defense and block test

#### 3.4.1 Defense

**Figure 4:**
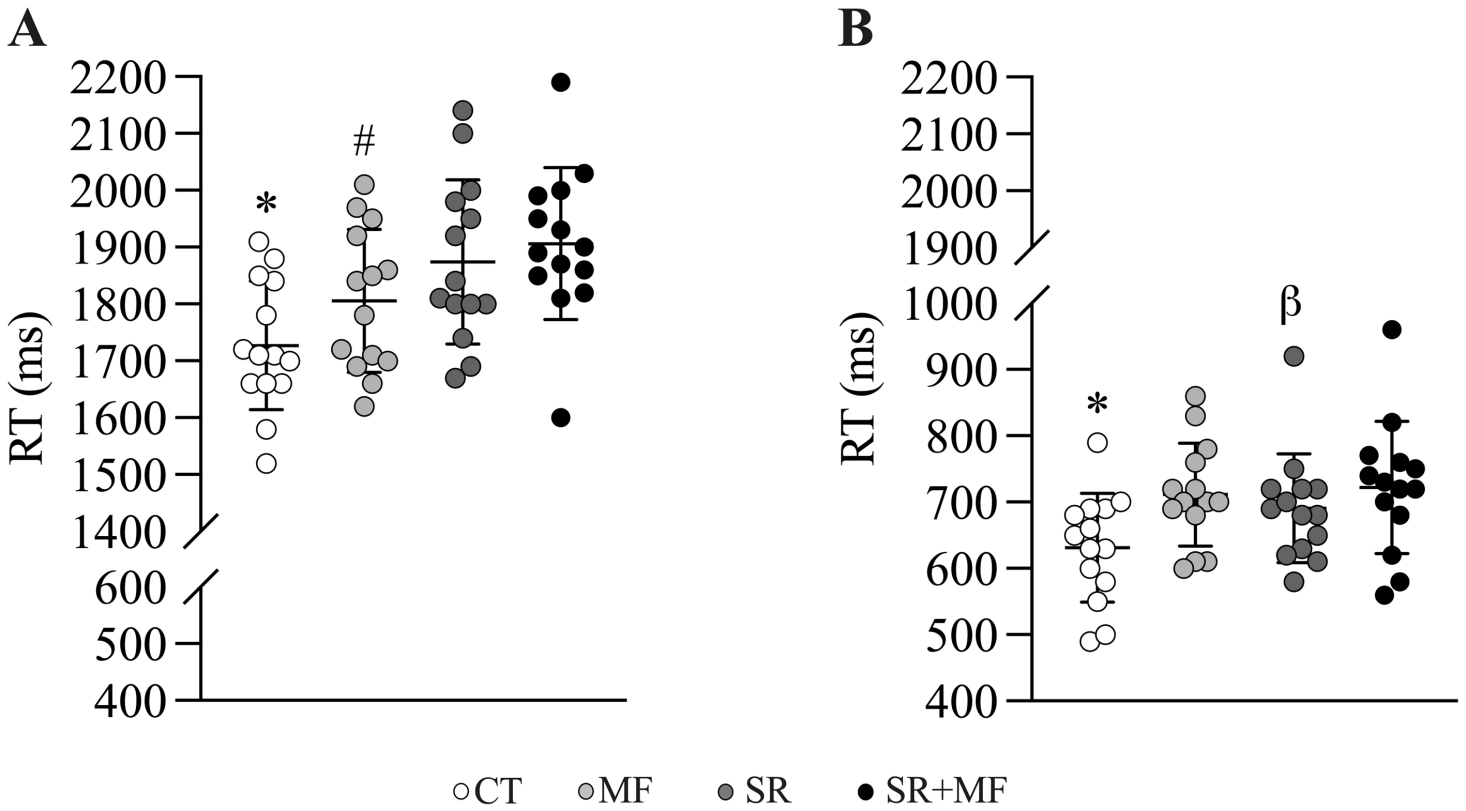
Perceptual-cognitive responses to the visuomotor defense **(A)** and block **(B)** tests in all experimental conditions. RT, reaction time; CT, control; MF, mental fatigue; SR, sleep restriction; SR + MF, sleep restriction + mental fatigue. ^*^Compared to all experimental conditions. ^#^Compared to SR and SR + MF experimental conditions. *β* compared to both MF and SR + MF experimental conditions.

A condition effect [*X*^2^_(3,39)_ = 212.2; *p* < 0.001] was observed (Figure 4). Participants in the CT condition presented better scores compared to MF [CT vs. MF: 1727.0 ± 113.0 ms vs. 1806.0 ± 126.0 ms; CI95%_diff_ = −80.6 (−105.0 to −56.0); *p* < 0.001], SR [CT vs. SR: 1727.0 ± 113.0 ms vs. 1874.0 ± 145.0 ms; CI95%_diff_ = −151.8 (−195.0 to −108.4); *p* < 0.001], and SR + MF [CT vs. SR + MF: 1727.0 ± 113.0 ms vs. 1906.0 ± 133.0 ms; CI95%_diff_ = −182.2 (−228.0 to −136.0); *p* < 0.001]. Additionally, among the experimental conditions, SR [SR vs. MF: 1874.0 ± 145.0 ms vs. 1806.0 ± 126.0 ms; CI95%_diff_ = 67.0 (3.04 to 110.1); *p* = 0.002] and SR + MF [SR + MF vs. MF: 1906.0 ± 133.0 ms vs. 1806.0 ± 126.0 ms; CI95%_diff_ = 101.6 (32.7 to 170.5); *p* < 0.001] presented different values compared to MF. However, between SR and SR + MF the values were similar [SR vs. SR + MF: 1874.0 ± 145.0 ms vs. 1906.0 ± 133.0 ms; CI95%_diff_ = −30.7 (−42.9 to 104.4); *p* = 0.41].

#### 3.4.2 Block

We observed a condition effect [*X*^2^_(3,39)_ = 104.0; *p* < 0.001] for the visuomotor block test (Figure 4). Similarly to the defense test, CT condition presented better scores compared to MF [CT vs. MF: 631.0 ± 82.2 ms vs. 711.0 ± 77.5 ms; CI95%_diff_ = −82.6 (−102.1 to −63.1); *p* < 0.001], SR [CT vs. SR: 631.0 ± 82.2 ms vs. 691.0 ± 82.3 ms; CI95%_diff_ = −61.2 (−80.4 to −42.1); *p* < 0.001], and SR + MF [CT vs. SR + MF: 631.0 ± 82.2 ms vs. 722.0 ± 100.0 ms; CI95%_diff_ = −88.0 (−107.6 to −68.3); *p* < 0.001]. Interestingly, SR presented better scores compared to MF [SR vs. MF: 691.0 ± 82.3 ms vs. 711.0 ± 77.5 ms; CI95%_diff_ = −21.4 (−41.8 to −0.96); *p* = 0.04] and SR + MF [SR vs. SR + MF: 691.0 ± 82.3 ms vs. 722.0 ± 100.0 ms; CI95%_diff_ = −26.7 (−47.1 to −6.4); *p* = 0.01]. However, no differences were found between MF and SR + MF [MF vs. SR + MF: 711.0 ± 77.5 ms vs. 722.0 ± 100.0 ms; CI95%_diff_ = −5.4 (−26.0 to 15.3); *p* = 0.61].

## 4 Discussion

This study aimed to analyze the isolated and combined effects of MF and SR on perceptual-cognitive performance in youth trained beach volleyball players. The main findings suggest that both MF and SR, individually and in combination, negatively impact RT in the defense and block visuomotor tests in this population.

An adapted version of the Stroop task (Stroop, 1935) induced MF in this study by using only incongruent stimuli and a “switch” function to increase cognitive load, with task duration individualized for each participant. MF was successfully induced, as evidenced by increased VAS. Also, athletes who underwent SR + MF showed compromised accuracy at the end of the Stroop task, likely due to reduced motivation (Milyavskaya et al., 2021) and effort which may cause a decreased effort to perform the task in the presence of an unattractive reward.

To individualize the cognitive demand load, a double-check 70 mm threshold at VAS was used with no time restriction on the task. This configuration differed from previous studies with time restrictions (Gantois et al., 2020; Shaabani et al., 2020; Vogt et al., 2018; Moreira et al., 2018; Van Cutsem et al., 2019; Filipas et al., 2021b), where athletes reached between 44 and 70 mm in the VAS when submitted to the Stroop task (Van Cutsem et al., 2019; Filipas et al., 2021b; Kosack et al., 2020). Adopting the 70 mm double-check in the VAS with no time restriction to end the cognitive-demand task (30 min as minimal duration) was deemed suitable to individualize the cognitive load, given that 70 mm was the maximum observed in a previous study (Filipas et al., 2021b), and a 30 min duration of the cognitive-demand task is typical in previous studies (Van Cutsem et al., 2017). MF induction in this study lasted, on average, 55 min.

The trained athletes in this study exhibited relatively low sleep efficiency (<85%). Compared to elite athletes, they appear to have less efficient sleep (Miles et al., 2022), possibly due to the growing awareness of sleep’s role in performance and the more controlled environments in which elite athletes train. Additionally, the athletes in this study may be more affected by both sport-related and non-sport factors that contribute to sleep disturbances, possibly due to the challenges recreational sports face in assessing athletes’ sleep and the limited guidance and monitoring provided by coaches regarding sleep hygiene (Randell et al., 2021). Higher sleepiness and impaired accuracy were observed post-MF induction, which could be attributed to the self-control demands of the Stroop task leading to boredom (Thompson et al., 2020), that is associated with less effort to complete tasks and feelings of frustration and fatigue (Bieleke et al., 2021). Boredom can impair sustained attention, potentially related to the nucleus accumbens region of the brain (Oishi et al., 2017). Additionally, volunteers under experimental conditions with SR were sleepier than those under habitual sleep conditions which corroborate with previous studies (Van Dongen et al., 2003; Skurvydas et al., 2020; Pallesen et al., 2017; Watson, 2017; Gattoni, 2019; Otmani et al., 2005). Also, sleepiness and MF were higher in volunteers who underwent SR conditions regardless of the time-point analysis, possibly due to the endogenous circadian regulation system (Goel et al., 2013; Reilly, 1990) and the time-of-day effect on mental fatigue (Hockey, 2013; Matthews et al., 2012; Gunzelmann et al., 2011; Wiehler et al., 2022; McCormick et al., 2012).

The results demonstrated that both SR and MF negatively impacted performance in the perceptual-cognitive tests, with increased RT compared to the CT condition. In defense test, the negative impact of SR and SR + MF was greater than MF, indicating that SR impairs athletes’ ability to quickly process stimuli and react and suggesting an additive effect of SR and MF on RT to the defense test. Additionally, regarding the block test, MF caused greater impairment than SR on blocking performance. Still, no significant difference was found between MF and SR + MF, suggesting SR may overshadow the effects of MF. The block test primarily assesses RT, while the defense test focuses on movement speed after RT. These distinct dimensions of motor performance reflect the cognitive and motor demands specific to beach volleyball, where rapid reactions and effective movement in unpredictable sand conditions are both critical (Cañal-Bruland et al., 2011). The differential impact of SR and MF on these tests highlights the complex interaction between cognitive and physical fatigue in beach volleyball athletes’ performance.

Given that, these findings align with previous studies that have observed adverse effects of SD/SR on perceptual-cognitive performance in athletes (Charest and Grandner, 2020; Fullagar et al., 2023) and the effects of MF alone (Cao et al., 2022; Habay et al., 2021a). Besides, studies that assessed perceptual-cognitive performance from the real context of sports do so from the analysis of video games (Coutinho et al., 2017; Fortes et al., 2022b) or in a simulated soccer game context (Gantois et al., 2020; Fortes et al., 2019; Fortes et al., 2020), where the endurance component can negatively influence performance. It is worth noting that the visuomotor defense test proposed in this study, although lasting an average of 3 min and 5 s, was performed intermittently.

Studies have found that badminton (Van Cutsem et al., 2019; Van Cutsem et al., 2020), table tennis (Habay et al., 2021b), and basketball athletes (Faro et al., 2022) demonstrate impaired perceptual-cognitive performance under MF in sport-related simulations using visuomotor-led light tests. These studies involved athletes with an average age of 24–25 years and employed more complex visual stimuli using six to eight LED lights. In contrast, our study assessed athletes aged 16–20 years and used simpler stimuli with three LED lights, which may explain why impairments under the MF condition were less pronounced compared to SR. In this sense, previous research suggests that younger athletes may be more resistant to MF than adult recreational athletes, which could reflect attenuated impairments in performance (Filipas et al., 2018). This is possibly due to the development of brain areas related to inhibitory response, such as the frontal lobe and anterior cingulate cortex, which develop considerably from 12 to 17 years of age (Romine and Reynolds, 2005). However, adults may have greater activation of brain regions such as the prefrontal, anterior cingulate and parietal cortex when performing cognitive tasks (Adleman et al., 2002), resulting in reduced impairments over time in younger individuals who have reduced accessibility to regions or computational skills that support complex behaviors (Luna et al., 2010).

Studies have shown that SR can negatively impact perceptual-cognitive performance in athletes. Tennis players’ serve accuracy (Reyner and Horne, 2013), handball goalkeepers’ RT, selective and constant attention (Jarraya et al., 2014), dart throwing players’ accuracy and consistency (Edwards and Waterhouse, 2009), and the frequency of racing drivers crossing track lines (Otmani et al., 2005) have been impaired due to SR. However, no study has evaluated the effects of SR on visuomotor stimuli tests that reproduce the ecological context of sports. Given that, this study aimed to fill this gap by examining beach volleyball athletes’ perceptual-cognitive performance.

Unlike previous studies which did not take into account athletes’ habitual sleep behavior (Jarraya et al., 2014; Otmani et al., 2005; Reyner and Horne, 2013; Edwards and Waterhouse, 2009), the present study incorporated this factor by restricting 50% of the athletes’ sleep time based on their habitual sleep patterns individually. The results support previous findings that SR impairs perceptual-cognitive performance in athletes and suggest that these impairments also affect tasks performed in real sports situations.

Perceptual-cognitive impairments caused by SD/SR have been attributed to the functioning of the prefrontal cortex, which is sensitive to cortical responsiveness and attention in the presence of an interrupted sleep state and increases the likelihood of failure to perform intended actions (Doran et al., 2001; Grundgeiger et al., 2014). Studies have also shown that brain metabolism decreases when sleep duration is reduced compared to the waking state; this decline occurs in regions such as the thalamus, cerebellum, prefrontal, posterior parietal, and temporal cortex (Taber and Hurley, 2006; Drummond and Brown, 2001), which are associated with impairments in cognitive performance (Thomas et al., 2003).

### 4.1 Limitations and strengths

Our study has some limitations: (I) the lack of monitoring of volunteers’ subjective perception of effort during visuomotor and physical tests; (II) the absence of chronotype analysis to recommend optimal sleep times based on athletes’ chronotypes; (III) the non-ecological method used for MF induction; (IV) the lack of measurements for temperature, humidity, diet, and hydration of the volunteers; (V) the absence of cross-cultural validation for the Karolinska Sleepiness Scale; (VI) the inclusion of only young beach volleyball athletes, limiting the generalizability of the findings to athletes of different age groups or competitive levels; and (VII) the heterogeneity of participants from different competitive levels. Regarding limitation 5, we performed a translation with the assistance of a native English professional, and volunteers subjected to SR exhibited greater sleepiness than those who were not.

Despite these limitations, the study presents several strengths: (I) the successful implementation of two experimental manipulations (MF and SR) resulting in impairments in MF’s subjective and behavioral assessments and greater sleepiness under SR conditions compared to no-SR conditions; (II) the individualization of cognitive load to induce MF, an approach rarely applied in sports science studies; (III) the evaluation of perceptual-cognitive performance in beach volleyball athletes using reproducible tests designed to simulate real-world scenarios; (IV) the individualized SR protocol (50% sleep reduction) based on habitual sleep behavior monitoring; and (V) the simultaneous analysis of SR and MF, both isolated and combined, as these conditions frequently occur in athletes’ daily routines and can negatively affect sports performance.

Future studies are required to (I) use ecological and controlled methods to induce MF (e.g., smartphones, video games) and individualize cognitive load and stimulus amount; (II) consider athletes’ chronotypes to determine the appropriate time for conducting SR (i.e., later at night or earlier in the morning); (III) evaluate athletic performance in real-context sports situations; and (IV) assess the combined repeated effects of SR and MF on athletes’ perceptual-cognitive performance.

## 5 Conclusion

The findings of this study reveal significant impairments in perceptual-cognitive performance under conditions of SR, MF, and their combination (SR + MF), as evidenced by slower RT in both the defense and block visuomotor tests compared to the CT condition. SR + MF caused greatest deficits compared to MF in the defense test and compared to SR in the block test, highlighting a cumulative effect when both stressors are combined. These results emphasize the need to manage these conditions to optimize athletic outcomes.

### 5.1 Practical recommendations

We recommend that athletes understand the potential impact of sleep on their performance and adopt behaviors that do not interfere with their usual sleep schedule. Coaches could provide sleep education to athletes to avoid sleep-distracting behaviors and encourage them to develop a routine for sleeping and waking up, avoid using electronic devices while lying down to sleep, and implement strategies to manage SR, such as taking short daytime naps (≈30 min), consuming caffeine, or combining both approaches (Fullagar et al., 2023; Nédélec et al., 2015; Jones et al., 2018; Romdhani et al., 2021). Coaches can support athletes in implementing sleep hygiene practices and countermeasures to mitigate the effects of SR (Walsh et al., 2021). Additionally, it is important to consider real-life scenarios where both SR and MF may co-occur, such as congested competition schedules, travel across time zones, academic demands in student-athletes, or social media demands (Fullagar et al., 2023). Understanding how these factors interact and influence in sleep patterns and mental fatigue could help coaches develop targeted strategies to mitigate their combined effects on perceptual-cognitive performance (Habay et al., 2021a; Fullagar et al., 2023; Walsh et al., 2021).

## Data Availability

The raw data supporting the conclusions of this article will be made available by the authors, without undue reservation.

## Footnotes

^1^ https://glimmpse.samplesizeshop.org/
