# Supplementary figures and images for: Mental fatigue and sleep restriction effects on perceptual-cognitive performance in trained beach volleyball athletes
Source: Front Psychol. 2025 May 9;16:1537482. doi: 10.3389/fpsyg.2025.1537482 (PMC12098282; doi:10.3389/fpsyg.2025.1537482)

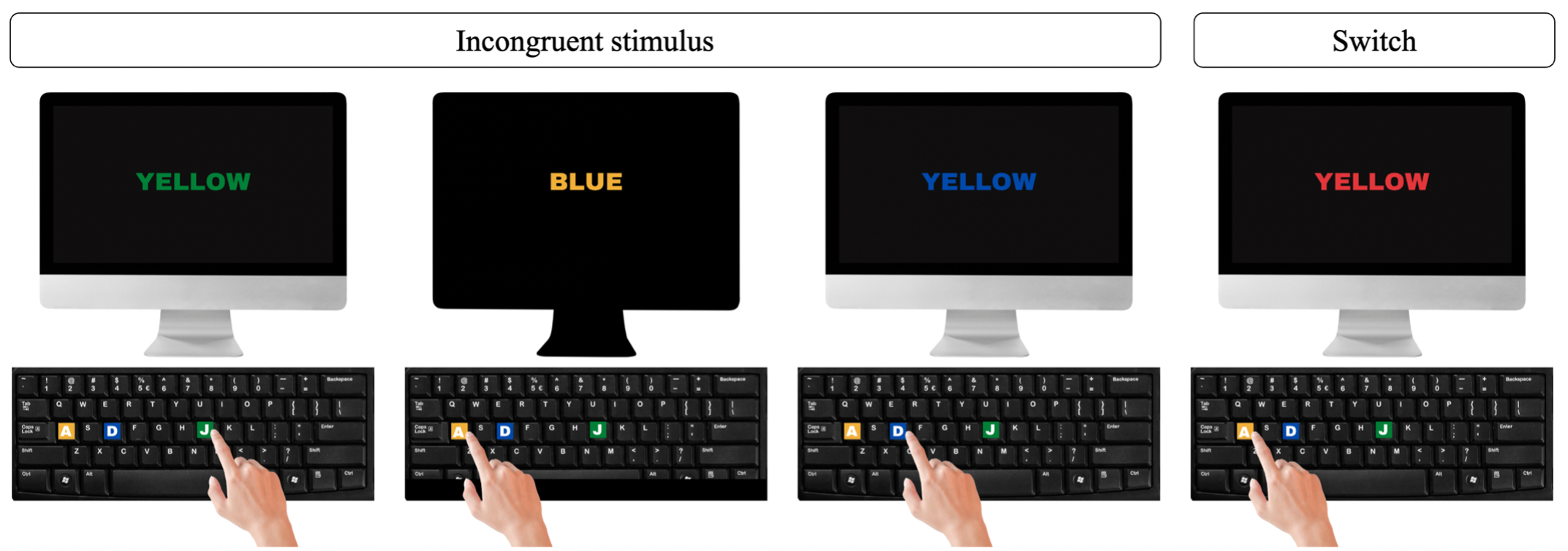

Supplement: Supplementary file 1 [file Image_1.JPEG]

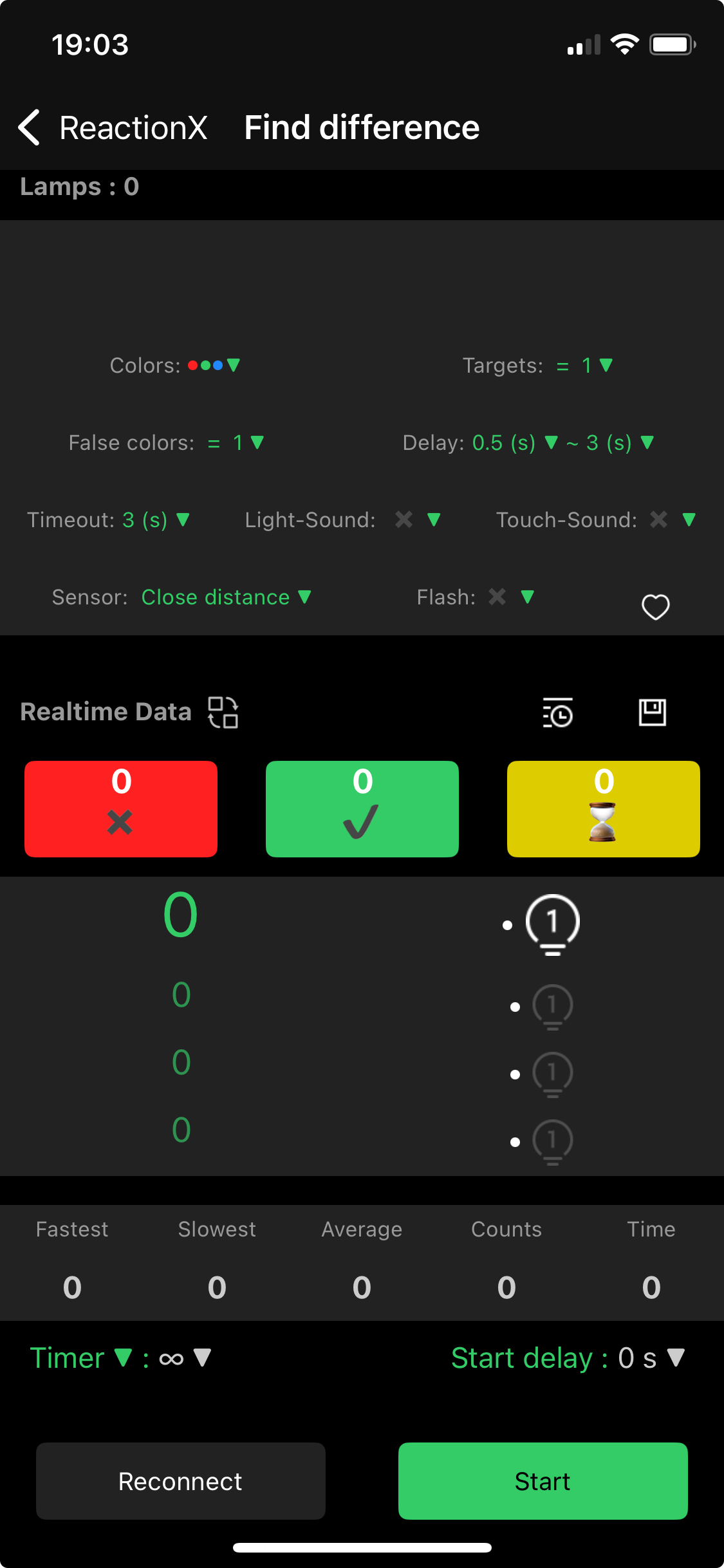

Supplement: Supplementary file 2 [file Image_2.JPEG]

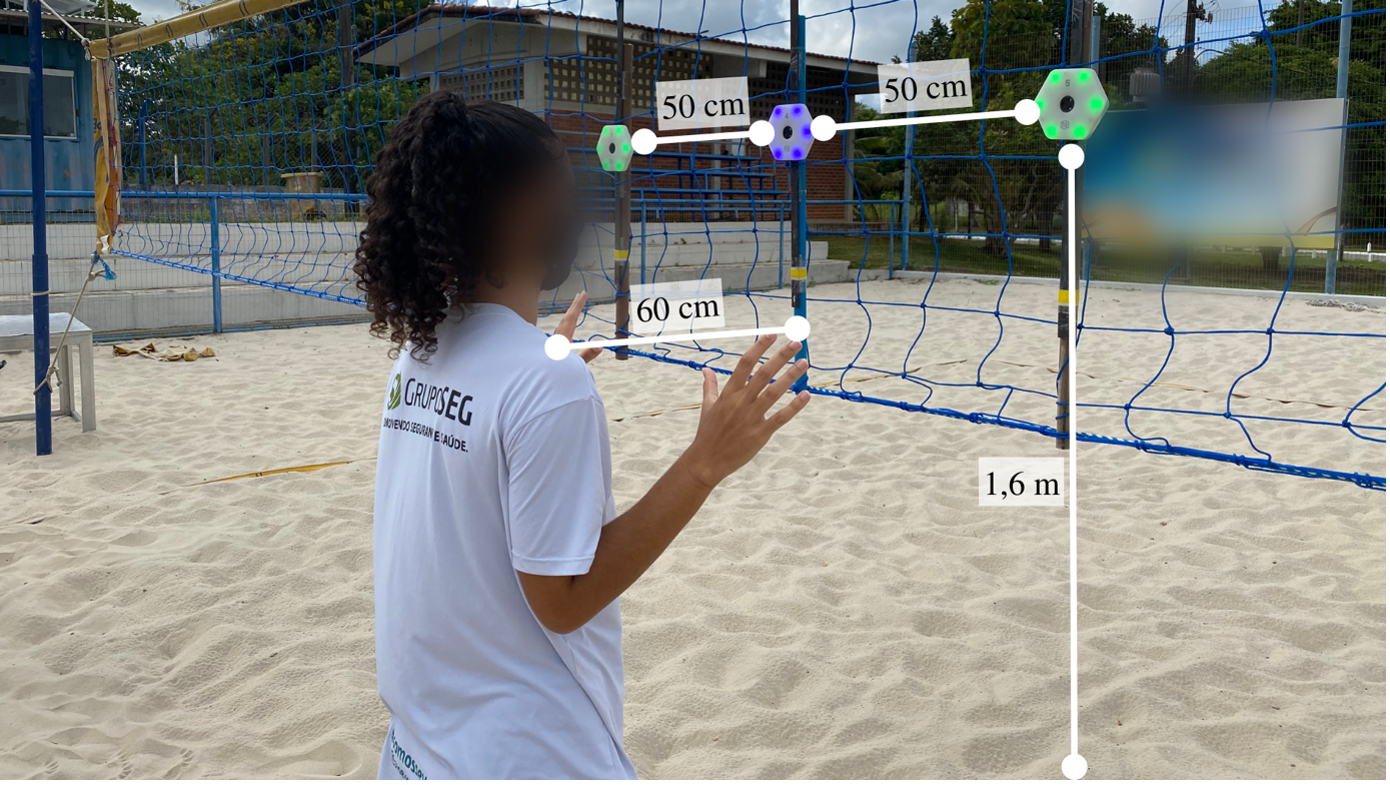

Supplement: Supplementary file 3 [file Image_3.JPEG]

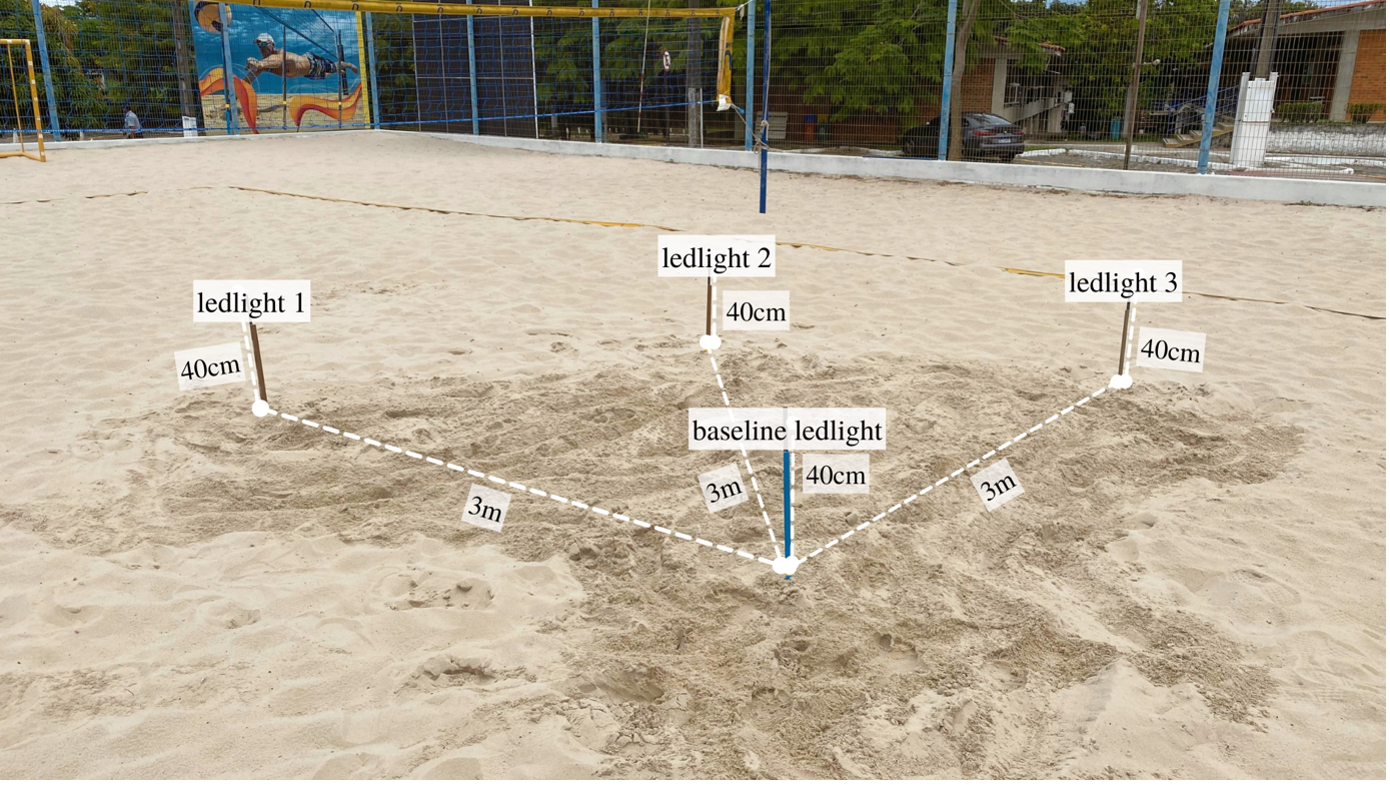

Supplement: Supplementary file 4 [file Image_4.JPEG]

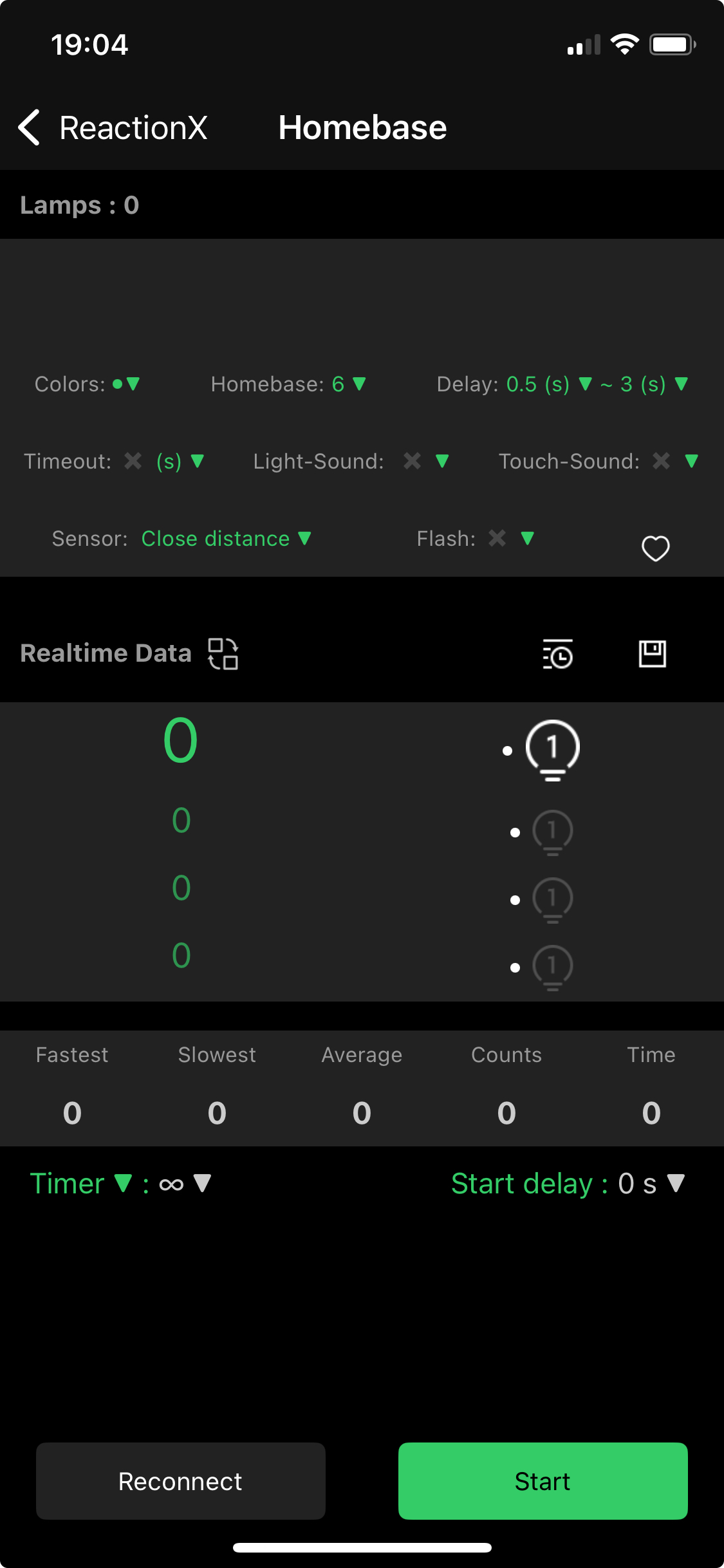

Supplement: Supplementary file 5 [file Image_5.JPEG]
